# DNA Barcoding of Metazoan Zooplankton Copepods from South Korea

**DOI:** 10.1371/journal.pone.0157307

**Published:** 2016-07-06

**Authors:** Su Youn Baek, Kuem Hee Jang, Eun Hwa Choi, Shi Hyun Ryu, Sang Ki Kim, Jin Hee Lee, Young Jin Lim, Jimin Lee, Jumin Jun, Myounghai Kwak, Young-Sup Lee, Jae-Sam Hwang, Balu Alagar Venmathi Maran, Cheon Young Chang, Il-Hoi Kim, Ui Wook Hwang

**Affiliations:** 1 School of Life Sciences, Graduate School, Kyungpook National University, Daegu, South Korea; 2 Department of Biology, Teachers College & Institute for Phylogenomics and Evolution, Kyungpook National University, Daegu, South Korea; 3 Freshwater Biodiversity Research Division, Nakdonggang National Institute of Biological Resources, Sangju, Gyeongsangbuk-do, Republic of Korea; 4 Marine Ecosystem Research Division, Korea Institute of Ocean Science and Technology, Ansan, South Korea; 5 Biological Resources Research Department, National Institute of Biological Resources, Incheon, South Korea; 6 Department of Agricultural Biology, National Academy of Agricultural Science, Rural Development Administration, Jeonju, Republic of Korea; 7 Department of Biological Science, College of Natural Sciences, Daegu University, Gyeongsan, South Korea; 8 Department of Biology, Gangneung-Wonju National University, Gangneung, Gangwon-Do,South Korea; National Taiwan Ocean University, TAIWAN

## Abstract

Copepods, small aquatic crustaceans, are the most abundant metazoan zooplankton and outnumber every other group of multicellular animals on earth. In spite of ecological and biological importance in aquatic environment, their morphological plasticity, originated from their various lifestyles and their incomparable capacity to adapt to a variety of environments, has made the identification of species challenging, even for expert taxonomists. Molecular approaches to species identification have allowed rapid detection, discrimination, and identification of cryptic or sibling species based on DNA sequence data. We examined sequence variation of a partial mitochondrial cytochrome *C* oxidase I gene (*COI*) from 133 copepod individuals collected from the Korean Peninsula, in order to identify and discriminate 94 copepod species covering six copepod orders of Calanoida, Cyclopoida, Harpacticoida, Monstrilloida, Poecilostomatoida and Siphonostomatoida. The results showed that there exists a clear gap with ca. 20 fold difference between the averages of within-specific sequence divergence (2.42%) and that of between-specific sequence divergence (42.79%) in *COI*, suggesting the plausible utility of this gene in delimitating copepod species. The results showed, with the *COI* barcoding data among 94 copepod species, that a copepod species could be distinguished from the others very clearly, only with four exceptions as followings: *Mesocyclops dissimilis*–*Mesocyclops pehpeiensis* (0.26% K2P distance in percent) and *Oithona davisae*–*Oithona similis* (1.1%) in Cyclopoida, *Ostrincola japonica*–*Pseudomyicola spinosus* (1.5%) in Poecilostomatoida, and *Hatschekia japonica*–*Caligus quadratus* (5.2%) in Siphonostomatoida. Thus, it strongly indicated that *COI* may be a useful tool in identifying various copepod species and make an initial progress toward the construction of a comprehensive DNA barcode database for copepods inhabiting the Korean Peninsula.

## Introduction

Copepods are one of the prevalent taxonomic groups among crustaceans, encompassing approximately 14,000 described species worldwide [1, 2, 3], of which about 695 species from 97 families have been known to occur in Korean waters (http://www.kbr.go.kr/home/find/find02001l.do). Their incomparable capability of adaptation to diverse environmental conditions has probably led to their extraordinary morphological and ecological diversity; as a consequence, copepod species are distributed throughout the world and found in nearly every kind of aquatic habitats [2, 4]. In addition, the diversity of copepod species is directly associated with maintaining natural resources as well as nourishing human life, since many of them numerically dominate most planktonic communities [1, 5], play a pivotal function in aquatic food webs [6], regulate global carbon cycle and climate [7–8] and live as endo- or ectoparasites in many aquatic animals [1, 4, 9]. Despite the ecological and economic significance, little is known about the number of copepod species on earth.

In recent years, because of their ecological importance, a lot of attention has been placed on the estimation of the biodiversity of this subclass Copepoda in marine and freshwater ecosystems [10–13]. The identification and classification of copepods have fundamentally been based on their morphological and anatomical characteristics [1, 2, 4]. However, such conventional ways may have some limitation in precisely estimating the abundance of copepod species in a certain environment, because they are time-consuming and necessitate special training or professional skills. Another difficulty may also be the existence of closely related taxa that are barely distinguishable [12, 14–16]. To make it more difficult, many of copepod species display morphological intraspecific variation corresponding to the habitat types [17]. Consequently, the application of a rapid and promising protocol for the species identification is critically needed for the estimation of copepod diversity.

Many different genetic markers have been considered to complement those conventional approaches. Mitochondrial cytochrome *C* oxidase subunit I gene (*COI*) is the gene offering the most efficient and accurate barcoding method for species-level identification in animal kingdom [18–21], though its efficiency is limited in taxa showing little nucleotide sequence diversity of mitochondrial DNA, such as scleractinian corals and calcarean sponges [22–24]. The partial *COI* barcoding region, which is ca. 600 bp in length, has been found valuable to reveal cryptic species that may not be possible to resolve the phylogenetic relationships in many copepods [12, 16, 25–27]. Numerous published studies for a variety of copepods have also proved the usefulness of *COI* in identifying species [28–30]. The *COI* gene is also effective in investigating phylogenetic relationship among species or higher taxa [27, 30–32]. Whereas *COI* has been analyzed from many calanoid and cyclopoid copepods, relatively limited genetic information is available for the remaining orders.

In the present study, the *COI* diversity was investigated from 133 individuals of 94 species of copepods representing six orders, Calanoida, Cyclopoida, Harpacticoida, Monstrilloida, Poecilostomatoida and Siphonostomatoida. Until now, extensive DNA barcoding study has never been done over the six copepod orders. Specifically, *COI* barcoding has never been attempted in the order Monstrilloida. Primary aims of this study are (i) to test whether *COI* is a sufficient and promising marker to identify various copepod species and (ii) to create preliminary progress towards the construction of a comprehensive DNA barcode database for identified specimens of copepods inhabiting the Korean Peninsula.

## Materials and Methods

### Sample collection

**Fig 1:**
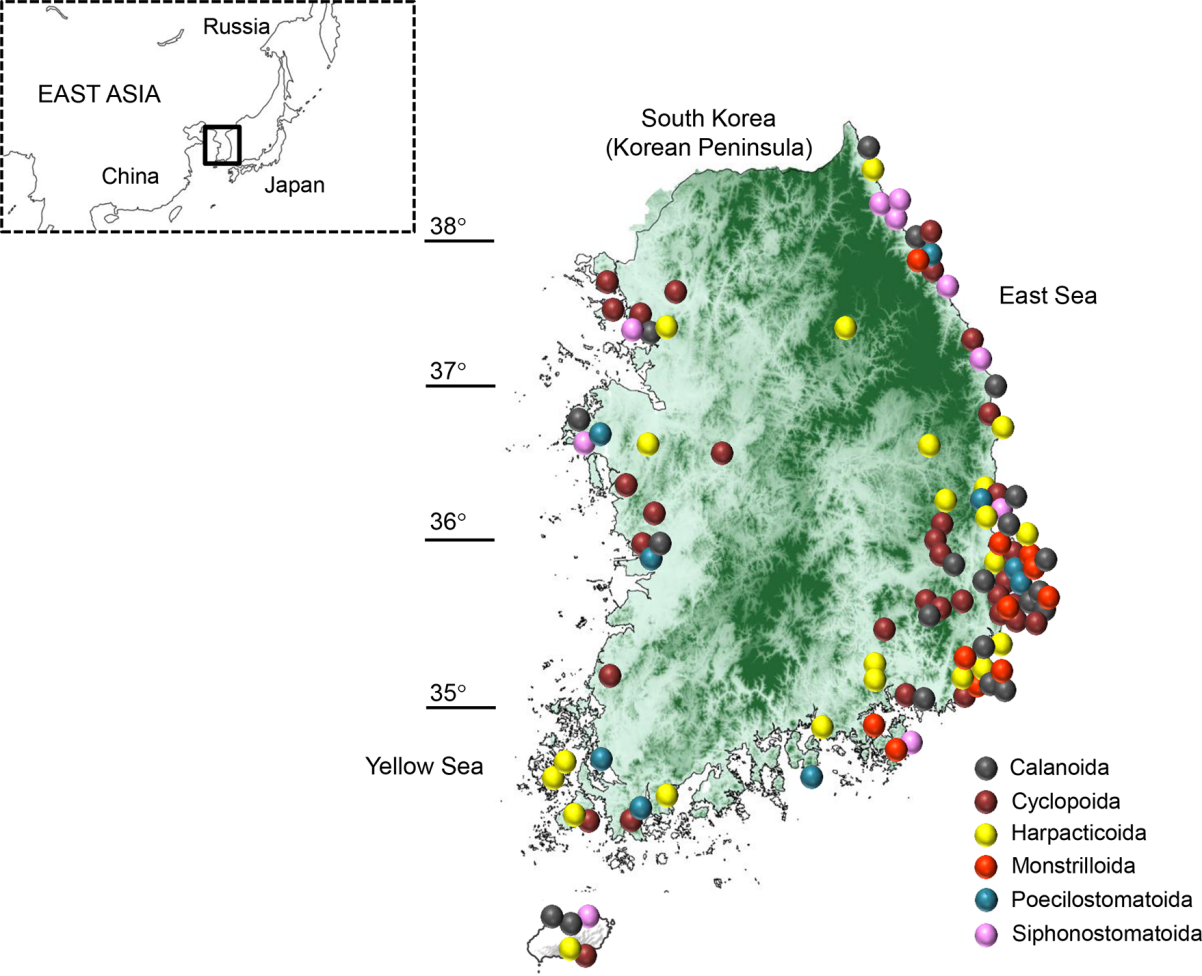
Collecting locations of 94 copepod species including the six orders used in the present study.

Specimens were collected from 2003 to 2014 across freshwater systems, coastal and oceanic areas on and around South Korea (Fig 1). Collection of every sample examined here did not require permission from government authorities, because copepods are an invertebrate animal, for which collecting regulations are not strictly controlled in South Korea. Nevertheless, we received permission from the Ministry of Environment of the Korean government for our sample collection in the present study. Individual specimens were carefully identified based on morphological characters. The entire bodies of all individuals were preserved in 95% ethanol. Species names, GenBank accession numbers and other characteristics of all taxa used in the present study are listed in Table 1.

**Table 1:** Summary of classification, species name, adult life style, collection locality, GPS, GenBank accession numbers, and used PCR primers for 133 samples covering 94 copepod species examined here.

Classification	Species	LS*	Voucher No.	Location	GPS	GenBank Acc. No.	PS**
**Order Calanoida**							
Acartiidae	*Acartia erythrea*	Fl	LEGO-CAL002-1	Buheung-ri, Namjeong-myeon, Yeongdeok-gun, Gyeongsangbuk-do	36.292274, 129.377329	KR048930	Ⅰ/Ⅱ
			LEGO-CAL002-2	Yonghan-ri, Heunghae-eup, Buk-gu, Pohang-si, Gyeongsangbuk-do	36.112617, 129.427250	KR048931	Ⅰ/Ⅱ
	*Acartia steueri*	Fl	LEGO-CAL005	Samjeong-ri, Guryongpo-eup, Nam-gu, Pohang-si, Gyeongsangbuk-do	36.003694, 129.571265	KR048932	Ⅰ/Ⅱ
			LEGO-CAL005-9	Gampo-ri, Gampo-eup, Gyeongju-si, Gyeongsangbuk-do	35.804109, 129.504398	KR048933	Ⅰ/Ⅱ
			LEGO-CAL005-10	Gyewon-ri, Janggi-myeon, Nam-gu, Pohang-si, Gyeongsangbuk-do	35.862180, 129.526584	KR048934	Ⅰ/Ⅱ
	*Acartia tsuensis*	Fl	LEGO-CAL007	Jindong-ri, Jindong-myeon, Masanhappo-gu, Changwon-si, Gyeongsangnam-do	35.152335, 128.611304	KR048935	Ⅲ/Ⅱ
			LEGO-CAL007-13	Jindong-ri, Jindong-myeon, Masanhappo-gu, Changwon-si, Gyeongsangnam-do	35.152335, 128.611304	KR048936	Ⅲ/Ⅱ
Calanidae	*Calanus sinicus*	Fl	LEGO-CAL019	Nampo-dong 1-ga, Jung-gu, Busan	35.096872, 129.032409	KR048947	Ⅵ/Ⅱ
			LEGO-CAL019-70	Jinhae-gu, Changwon-si, Gyeongsangnam-do	35.133799, 128.672114	KR048948	Ⅷ/Ⅸ
			LEGO-CAL019-71	Wollae-ri, Jangan-eup, Gijang-gun, Busan	35.327013, 129.280212	KR048949	Ⅵ/Ⅱ
Centropagidae	*Sinocalanus tenellus*	Fl	LEGO-CAL033-15	Daebudong-dong, Danwon-gu, Ansan-si, Gyeonggi-do	37.233211, 126.602035	KR048937	Ⅰ/Ⅱ
			LEGO-CAL033-16	Samsan-dong, Nam-gu, Ulsan	35.544860, 129.354529	KR048938	Ⅰ/Ⅱ
			LEGO-CAL033-17	Nampobangjoje-ro, Boryeong-si, Chungcheongnam-do	36.264025, 126.547897	KR048939	Ⅰ/Ⅱ
Diaptomidae	*Heliodiaptomus kikuchii*	Fl	LEGO-CAL038	Naeri-ri, Jillyang-eup, Gyeongsan-si, Gyeongsangbuk-do	35.896667, 128.846775	KR048940	Ⅳ/Ⅴ
	*Neodiaptomus schmackeri*	Fl	LEGO-CAL041	Sangnim-ri, Jillyang-eup, Gyeongsan-si, Gyeongsangbuk-do	35.908780, 128.830534	KR048941	Ⅰ/Ⅱ
			LEGO-CAL041-23	Neungcheon-ri, Yongmun-myeon, Yecheon-gun, Gyeongsangbuk-do	36.702354, 128.424865	KR048942	Ⅰ/Ⅱ
			LEGO-CAL041-24	Sinwol-ri, Geumho-eup, Yeongcheon-si, Gyeongsangbuk-do	35.939784, 128.901072	KR048943	Ⅰ/Ⅱ
			LEGO-CAL041-26	Osu-dong, Yeongcheon-si, Gyeongsangbuk-do	35.956514, 128.921268	KR048944	Ⅰ/Ⅱ
	*Sinodiaptomus sarsi*	Fl	LEGO-CAL042-27	Juhang-ri, Seo-myeon, Seocheon-gun, Chungcheongnam-do	36.155484, 126.571364	KR048945	Ⅳ/Ⅴ
	*Acanthodiaptomus pacificus*	Fl	LEGO-CAL037	Daeheul-ri, Jocheon-eup, Jeju-si, Jeju-do	33.468988, 126.667769	KR048946	Ⅲ/Ⅱ
			-	Japan (Makino and Tanabe, 2009)	-	AB494174	-
Paracalanidae	*Paracalanus parvus*	Fl	LEGO-CAL057	Yonghan-ri, Heunghae-eup, Buk-gu, Pohang-si, Gyeongsangbuk-do	36.112617, 129.427250	KR048950	Ⅷ/Ⅹ
			LEGO-CAL057-33	Sinchang-ri, Janghang-eup, Seocheon-gun, Chungcheongnam-do	36.007095, 126.692016	KR048951	Ⅵ/Ⅱ
			LEGO-CAL057-34	Geumjin-ri, Ganggu-myeon, Yeongdeok-gun, Gyeongsangbuk-do	36.376379, 129.401393	KR048952	Ⅲ/Ⅱ
Classificarion	Species	LS*	Voucher No.	Location	GPS	GenBank Acc. No.	PS**
Pseudodiaptomidae	*Pseudodiaptomus inopinus*	Fl	LEGO-CAL063	Gosan-ri, Hangyeong-myeon, Jeju-si, Jeju-do	33.307396, 126.163262	KR048953	Ⅷ/Ⅸ
			LEGO-CAL063-44	Daebudong-dong, Danwon-gu, Ansan-si, Gyeonggi-do	37.229179, 126.600490	KR048954	Ⅷ/Ⅸ
			LEGO-CAL063-45	Sindu-ri, Wonbuk-myeon, Taean-gun, Chungcheongnam-do	36.836185, 126.182155	KR048955	Ⅷ/Ⅸ
	*Pseudodiaptomus marinus*	Fl	LEGO-CAL066	Sindu-ri, Wonbuk-myeon, Taean-gun, Chungcheongnam-do	36.836185, 126.182155	KR048956	Ⅲ/Ⅱ
			LEGO-CAL066-50	Yonghan-ri, Heunghae-eup, Buk-gu, Pohang-si, Gyeongsangbuk-do	36.112305, 129.428881	KR048957	Ⅰ/Ⅱ
			LEGO-CAL066-51	Wollae-ri, Jangan-eup, Gijang-gun, Busan	35.327013, 129.280212	KR048958	Ⅲ/Ⅱ
	*Pseudodiaptomus nihonkaiensis*	Fl	LEGO-CAL067	Gyewon-ri, Janggi-myeon, Nam-gu, Pohang-si, Gyeongsangbuk-do	35.862180, 129.526584	KR048959	Ⅲ/Ⅱ
			-	Korea (Eyun et al. 2007)	-	AF536519	-
Temoridae	*Eurytemora affinis*	Fl	LEGO-CAL077	Namdaecheon-ro, Seo-myeon, Yangyang-gun, Gangwon-do	38.032633, 128.601820	KR048960	Ⅲ/Ⅱ
	*Eurytemora pacifica*	Fl	LEGO-CAL078-59	Gampo-ri, Gampo-eup, Gyeongju-si, Gyeongsangbuk-do	35.808234, 129.504698	KR048961	Ⅵ/Ⅱ
			LEGO-CAL078	Yonghan-ri, Heunghae-eup, Buk-gu, Pohang-si, Gyeongsangbuk-do	36.112166, 129.428881	KR048962	Ⅲ/Ⅱ
			LEGO-CAL078-61	Joyang-dong, Sokcho-si, Gangwon-do	38.193728, 128.601078	KR048963	Ⅲ/Ⅱ
	*Temora turbinate*	Fl	LEGO-CAL081	Nampo-dong 1-ga, Jung-gu, Busan	35.096872, 129.032409	KR048964	Ⅲ/Ⅱ
			LEGO-CAL081-63	Nambumin-dong, Seo-gu, Busan	35.092961, 129.025250	KR048965	Ⅰ/Ⅱ
			LEGO-CAL081-64	Wollae-ri, Jangan-eup, Gijang-gun, Busan	35.327013, 129.280212	KR048966	Ⅰ/Ⅱ
**Order Cyclopoida**							
Cyclopidae	*Cyclops kikuchii*	Fl	LEGO-CYC007	Sangnim-ri, Jillyang-eup, Gyeongsan-si, Gyeongsangbuk-do	35.908780, 128.830534	KR048967	Ⅰ/Ⅱ
	*Diacyclops bicuspidatus*	Fl	LEGO-CYC010	Pyeongsa-ri, Jillyang-eup, Gyeongsan-si, Gyeongsangbuk-do	35.898130, 128.856595	KR048968	Ⅲ/Ⅱ
	*Macrocyclops albidus*	Fl	LEGO-CYC017	Indong-ri, Gangdong-myeon, Gyeongju-si, Gyeongsangbuk-do	35.988227, 129.255676	KR048969	Ⅲ/Ⅱ
			-	Mexico (Prosser et al. 2013)	-	KC617060	-
			-	Mexico (Prosser et al. 2013)	-	KC617660	-
	*Megacyclops viridis*	Fl	LEGO-CYC019	Pyeongsa-ri, Jillyang-eup, Gyeongsan-si, Gyeongsangbuk-do	35.898130, 128.856595	KR048970	Ⅲ/Ⅱ
			LEGO-CYC019-72	Daegudae-ro, Gyeongsan-si, Gyeongsangbuk-do	35.898210, 128.843872	KR048971	Ⅵ/Ⅱ
			LEGO-CYC019-71	Juhang-ri, Seo-myeon, Seocheon-gun, Chungcheongnam-do	36.156038, 126.567802	KR048972	Ⅵ/Ⅴ
	*Mesocyclops pehpeiensis*	Fl	LEGO-CYC021-77	Naeri-ri, Jillyang-eup, Gyeongsan-si, Gyeongsangbuk-do	35.896667, 128.846775	KR048973	Ⅰ/Ⅱ
			-	Taiwan (Unpublished)	-	KJ020571	-
	*Mesocyclops dissimilis*	Fl	LEGO-CYC020-83	Naeri-ri, Jillyang-eup, Gyeongsan-si, Gyeongsangbuk-do	35.896667, 128.846775	KR048974	Ⅵ/Ⅱ
	*Acanthocyclops vernalis*	Fl	LEGO-CYC040	Ogok-dong, Gangseo-gu, Seoul	37.556788, 126.766500	KR048975	Ⅷ/Ⅹ
Classification	Species	LS*	Voucher No.	Location	GPS	GenBank Acc. No.	PS**
	*Apocyclops borneoensis*	Fl	LEGO-CYC046	Unseo-dong, Jung-gu, Incheon	37.422974, 126.426755	KR048976	Ⅵ/Ⅱ
	*Halicyclops itohi*	Fl	LEGO-CYC016	Hanja-ri, Hwangsan-myeon, Haenam-gun, Jeollanam-do	34.545315, 126.432664	KR048977	Ⅰ/Ⅶ
	*Paracyclops fimbriatus*	Fl	LEGO-CYC023	Saekdal-dong, Seogwipo-si, Jeju-do	33.244290, 126.405785	KR048978	Ⅲ/Ⅱ
	*Tropocyclops setulifer*	Fl	LEGO-CYC039	Seongnyugul-ro, Geunnam-myeon, Uljin-gun, Gyeongsangbuk-do	36.956665, 129.379810	KR048979	Ⅷ/Ⅸ
Notodelphyidae	*Bonnierilla curvicaudata*	Ec	LEGO-CYC028	Sacheonjin-ri, Sacheon-myeon, Gangneung-si, Gangwon-do	37.837785, 128.877136	KR048980	Ⅰ/Ⅱ
			LEGO-CYC028-107	Songjeong-dong, Gangneung-si, Gangwon-do	37.772045, 128.929185	KR048981	Ⅷ/Ⅸ
			LEGO-CYC028-108	Namae-ri, Hyeonnam-myeon, Yangyang-gun, Gangwon-do	37.950192, 128.776801	KR048982	Ⅵ/Ⅱ
	*Doropygus rigidus*	Ec	LEGO-CYC031	Gallam-ri, Wondeok-eup, Samcheok-si, Gangwon-do	37.263509, 129.323927	KR048983	Ⅵ/Ⅱ
	*Lonchidiopsis hartmeyeri*	Ec	LEGO-CYC033	Geumjin-ri, Okgye-myeon, Gangneung-si, Gangwon-do	37.642461, 129.043641	KR048984	Ⅲ/Ⅱ
	*Pachypygus curvatus*	Ec	LEGO-CYC034	Sin-ri, Sinji-myeon, Wando-gun, Jeollanam-do	34.334344, 126.800075	KR048985	Ⅲ/Ⅱ
Oithonidae	*Oithona similis*	Fl	LEGO-CYC036-117	Yonghan-ri, Heunghae-eup, Buk-gu, Pohang-si, Gyeongsangbuk-do	36.112305, 129.428881	KR048986	Ⅳ/Ⅴ
			LEGO-CYC036	Wollae-ri, Jangan-eup, Gijang-gun, Busan	35.327013, 129.280212	KR048987	Ⅲ/Ⅱ
	*Oithona davisae*	Fl	LEGO-CYC035	Samsan-dong, Nam-gu, Ulsan	35.544755, 129.354915	KR048988	Ⅰ/Ⅱ
**Order Harpacticoida**							
Ameiridae	*Nitokra spinipes*	Fl/B	LEGO-HAR003	Jinha-ri, Seosaeng-myeon, Ulju-gun, Ulsan	35.382490, 129.345288	KR049004	Ⅰ/Ⅱ
	*Nitokra lacustris*	Fl/B	LEGO-HAR002	Wollae-ri, Jangan-eup, Gijang-gun, Busan	35.327013, 129.280212	KR049005	Ⅳ/Ⅴ
Canthocamptidae	*Canthocamptus kitaurensis*	Fl/B	LEGO-HAR010	Ahwa-ri, Seo-myeon, Gyeongju-si, Gyeongsangbuk-do	35.890030, 129.044042	KR049006	Ⅰ/Ⅱ
Dactylopusiidae	*Dactylopusia pauciarticulata*	Fl/B	LEGO-HAR015	Gisamun-ri, Hyeonbuk-myeon, Yangyang-gun, Gangwon-do	38.006289, 128.731514	KR049007	Ⅳ/Ⅴ
Darcythompsoniidae	*Leptocaris brevicornis*	As	LEGO-HAR017	Dadae-dong, Saha-gu, Busan	35.061526, 128.956287	KR049008	Ⅵ/Ⅱ
Harpacticidae	*Tigriopus japonicas*	Fl/B	LEGO-HAR023-94	Manheung-dong, Yeosu-si, Jeollanam-do	34.773827, 127.742034	KR049009	Ⅳ/Ⅴ
			LEGO-HAR023-95	Seongsan-eup, Seogwipo-si, Jeju-do	33.373084, 126.872241	KR049010	Ⅳ/Ⅴ
	*Harpacticus uniremis*	Fl/B	LEGO-HAR050	Myeongchon-dong, Buk-gu, Ulsan	35.547631, 129.357244	KR049016	Ⅵ/Ⅱ
Laophontidae	*Paralaophonte congenera*	As	LEGO-HAR027	Jinha-ri, Seosaeng-myeon, Ulju-gun, Ulsan	35.382490, 129.345288	KR049011	Ⅰ/Ⅱ
Longipediidae	*Longipedia kikuchii*	Fl/B	LEGO-HAR029	Yonghan-ri, Heunghae-eup, Buk-gu, Pohang-si, Gyeongsangbuk-do	36.112305, 129.428881	KR049012	Ⅰ/Ⅱ
Miraciidae	*Diosaccus ezoensis*	As	LEGO-HAR032	Nampo-dong 1-ga, Jung-gu, Busan	35.096872, 129.032409	KR049013	Ⅵ/Ⅴ
Thalestridae	*Eudactylopus spectabilis*	Fl/B	LEGO-HAR041	Nampo-dong 1-ga, Jung-gu, Busan	35.096872, 129.032409	KR049015	Ⅷ/Ⅹ
Tisbidae	*Tisbe* sp.	Fl/B	LEGO-HAR039	Hwadang-ri, Georyu-myeon, Goseong-gun, Gyeongsangnam-do	34.984309, 128.428012	KR049014	Ⅵ/Ⅱ
Classification	Species	LS*	Voucher No.	Location	GPS	GenBank Acc. No.	PS**
**Order Monstrilloida**							
Monstrillidae	*Cymbasoma* sp.	Fl	LEGO-MON002	Namhang-ro, Yeongdo-gu, Busan	35.089018, 129.036041	KR048989	Ⅵ/Ⅱ
	*Cymbasoma reticulatum*	Fl	LEGO-MON001-11	Yangpo-ri, Janggi-myeon, Nam-gu, Pohang-si, Gyeongsangbuk-do	35.876949, 129.516774	KR048990	Ⅰ/Ⅱ
			LEGO-MON001-13	Heunghae-eup, Buk-gu, Pohang-si, Gyeongsangbuk-do	36.112305, 129.428881	KR048991	Ⅳ/Ⅴ
	*Monstrilla hamatapex*	Fl	LEGO-MON005-3	Gyewon-ri, Janggi-myeon, Nam-gu, Pohang-si, Gyeongsangbuk-do	35.870961, 129.530060	KR048992	Ⅳ/Ⅴ
			LEGO-MON005-5	Yonghan-ri, Heunghae-eup, Buk-gu, Pohang-si, Gyeongsangbuk-do	36.112305, 129.428881	KR048993	Ⅳ/Ⅱ
			LEGO-MON005	Samjeong-ri, Guryongpo-eup, Nam-gu, Pohang-si, Gyeongsangbuk-do	36.004319, 129.574870	KR048994	Ⅰ/Ⅱ
	*Monstrilla* sp.	Fl	LEGO-MON006-7	Wollae-ri, Jangan-eup, Gijang-gun, Busan	35.327013, 129.280212	KR048995	Ⅰ/Ⅱ
			LEGO-MON006-8	Jinha-ri, Seosaeng-myeon, Ulju-gun, Ulsan	35.382490, 129.345288	KR048996	Ⅰ/Ⅱ
	*Monstrilla* sp.3	Fl	LEGO-MON008-17	Honghyeon-ri, Nam-myeon, Namhae-gun, Gyeongsangnam-do	34.748376, 127.909712	KR048997	Ⅰ/Ⅱ
			LEGO-MON008	Songjeong-ri, Mijo-myeon, Namhae-gun, Gyeongsangnam-do	34.733785, 128.038619	KR048998	Ⅰ/Ⅱ
	*Monstrilla* sp.4	Fl	LEGO-MON009	Yonghan-ri, Heunghae-eup, Buk-gu, Pohang-si, Gyeongsangbuk-do	36.112305, 129.428881	KR048999	Ⅵ/Ⅱ
	*Monstrillopsis* sp.	Fl	LEGO-MON010-8	Namhang-ro, Yeongdo-gu, Busan	35.089018, 129.036041	KR049000	Ⅵ/Ⅱ
	*Monstrillopsis* sp.2	Fl	LEGO-MON011	Korea Maritime Univ., Dongsam 2-dong, Yeongdo-gu, Busan	35.077358, 129.087972	KR049001	Ⅳ/Ⅴ
	*Maemonstrilla simplex*	Fl	LEGO-MON015-9	Korea Maritime Univ., Dongsam 2-dong, Yeongdo-gu, Busan	35.077358, 129.087972	KR049002	Ⅵ/Ⅱ
			LEGO-MON015-10	Taejong-ro, Yeongdo-gu, Busan	35.077358, 129.087972	KR049003	Ⅵ/Ⅱ
**Order Poecilostomatoida**							
Bomolochidae	*Bomolochus bellones*	Ec	LEGO-POE041	Ganggu-ri, Ganggu-myeon, Yeongdeok-gun, Gyeongsangbuk-do	36.359633, 129.388916	KR049017	Ⅷ/Ⅹ
	*Bomolochus decapteri*	Ec	LEGO-POE001	Sacheonjin-ri, Sacheon-myeon, Gangneung-si, Gangwon-do	37.834125, 128.876106	KR049018	Ⅳ/Ⅴ
	*Nothobomolochus thambus*	Ec	LEGO-POE002	Gumi-dong, Donghae-si, Gangwon-do	37.485592, 129.126643	KR049019	Ⅵ/Ⅱ
Chondracanthidae	*Acanthochondria spirigera*	Ec	LEGO-POE003	Jeonchon-ri, Gampo-eup, Gyeongju-si, Gyeongsangbuk-do	35.790757, 129.492552	KR049020	Ⅵ/Ⅱ
	*Acanthochondria tchangi*	Ec	LEGO-POE004	Jeongwang-dong, Siheung-si, Gyeonggi-do	37.329353, 126.673087	KR049021	Ⅵ/Ⅱ
	*Brachiochondria pinguis*	Ec	LEGO-POE005	Gyeokpo-ri, Byeonsan-myeon, Buan-gun, Jeollabuk-do	35.623545, 126.467718	KR049022	Ⅲ/Ⅱ
	*Chondracanthus distortus*	Ec	LEGO-POE006	Yangpo-ri, Janggi-myeon, Nam-gu, Pohang-si, Gyeongsangbuk-do	35.876949, 129.516774	KR049023	Ⅵ/Ⅱ
	*Chondracanthus zei*	Ec	LEGO-POE042	Yangpo-ri, Janggi-myeon, Nam-gu, Pohang-si, Gyeongsangbuk-do	35.876949, 129.516774	KR049033	Ⅵ/Ⅱ
Clausidiidae	*Hemicyclops ctenidis*	Ec	LEGO-POE008	Hyangho-ri, Jumunjin-eup, Gangneung-si, Gangwon-do	37.912963, 128.815505	KR049024	Ⅲ/Ⅱ
	*Hemicyclops gomsoensis*	Ec	LEGO-POE009	Yangpo-ri, Janggi-myeon, Nam-gu, Pohang-si, Gyeongsangbuk-do	35.876949, 129.516774	KR049025	Ⅲ/Ⅶ
Classification	Species	LS*	Voucher No.	Location	GPS	GenBank Acc. No.	PS**
	*Hemicyclops spinosus*	Ec	LEGO-POE011	Gwangjin-ri, Hyeonnam-myeon, Yangyang-gun, Gangwon-do	37.951220, 128.776601	KR049026	Ⅵ/Ⅴ
	*Hemicyclops tanakai*	Ec	LEGO-POE050	Songnim-ri, Janghang-eup, Seocheon-gun, Chungcheongnam-do	36.028871, 126.666395	KR049027	Ⅰ/Ⅱ
Clausiidae	*Clausia* sp.	Ec	LEGO-POE012-71	Gyeonso-dong, Gangneung-si, Gangwon-do	37.769060, 128.950915	KR049028	Ⅵ/Ⅴ
			LEGO-POE012	Gyeonso-dong, Gangneung-si, Gangwon-do	37.769060, 128.950915	KR049029	Ⅰ/Ⅱ
Ergasilidae	*Ergasilus* sp.	Ec	LEGO-POE13	Obong-ri, Jugwang-myeon, Goseong-gun, Gangwon-do	38.335302, 128.520282	KR049035	Ⅵ/Ⅶ
	*Ergasilus wilsoni*	Ec	LEGO-POE014	Obong-ri, Jugwang-myeon, Goseong-gun, Gangwon-do	38.335302, 128.520282	KR049036	Ⅵ/Ⅱ
	*Neoergasilus japonicus*	Ec	LEGO-POE015	Jukheon-dong, Gangneung-si, Gangwon-do	37.779466, 128.859372	KR049037	Ⅵ/Ⅴ
Taeniacanthidae	*Anchistrotos kojimensis*	Ec	LEGO-POE033	Nonhyeon-dong, Namdong-gu, Incheon	37.398895, 126.740576	KR049049	Ⅵ/Ⅱ
	*Taeniacanthus congeri*	Ec	LEGO-POE034	Seo-dong, Sacheon-si, Gyeongsangnam-do	34.926445, 128.068893	KR049030	Ⅲ/Ⅶ
			LEGO-POE034-1	Sinjindo-ri, Geunheung-myeon, Taean-gun, Chungcheongnam-do	36.682945, 126.138751	KR049031	Ⅲ/Ⅱ
	*Taeniacanthus yamagutii*	Ec	LEGO-POE035	Gyeokpo-ri, Byeonsan-myeon, Buan-gun, Jeollabuk-do	35.623545, 126.467718	KR049032	Ⅵ/Ⅱ
Lichomolgidae	*Synstellicola paracarens*	Ec	LEGO-POE021	Seung-eon-ri, Anmyeon-eup, Taean-gun, Chungcheongnam-do	36.597743, 126.323065	KR049034	Ⅵ/Ⅱ
	*Herrmannella dentata*	Ec	LEGO-POE017	Geumsong-ri, Samdong-myeon, Namhae-gun, Gyeongsangnam-do	34.830260, 128.011148	KR049038	Ⅵ/Ⅱ
	*Herrmannella hoonsooi*	Ec	LEGO-POE029	Seoho-dong, Tongyeong-si, Gyeongsangnam-do	34.839944, 128.418257	KR049039	Ⅵ/Ⅱ
	*Lichomolgus similis*	Ec	LEGO-POE037	Dueo-ri, Simwon-myeon, Gochang-gun, Jeollabuk-do	35.529463, 126.536684	KR049044	Ⅵ/Ⅱ
	*Modiolicola bifida*	Ec	LEGO-POE018	Yongjeong-ri, Hyeongyeong-myeon, Muan-gun, Jeollanam-do	35.049275, 126.379666	KR049040	Ⅵ/Ⅱ
	*Zygomolgus dentatus*	Ec	LEGO-POE022	Yangpo-ri, Janggi-myeon, Nam-gu, Pohang-si, Gyeongsangbuk-do	35.876949, 129.516774	KR049048	Ⅲ/Ⅱ
Myicolidae	*Ostrincola japonica*	Ec	LEGO-POE023	Hwayang-myeon, Yeosu-si, Jeollanam-do	34.709266, 127.619811	KR049041	Ⅵ/Ⅱ
	*Pseudomyicola spinosus*	Ec	LEGO-POE025-21	Geumjin-ri, Okgye-myeon, Gangneung-si, Gangwon-do	37.642461, 129.043641	KR049042	Ⅵ/Ⅱ
			LEGO-POE025	Gyeonso-dong, Gangneung-si, Gangwon-do	37.769060, 128.950915	KR049043	Ⅵ/Ⅱ
Rhynchomolgidae	*Critiomolgus vicinus*	Ec	LEGO-POE027	Seung-eon-ri, Anmyeon-eup, Taean-gun, Chungcheongnam-do	36.597743, 126.323065	KR049045	Ⅲ/Ⅱ
	*Zamolgus cavernularius*	Ec	LEGO-POE028-1	Daebubuk-dong, Danwon-gu, Ansan-si, Gyeonggi-do	37.229179, 126.600490	KR049047	Ⅲ/Ⅱ
			LEGO-POE028	Daebubuk-dong, Danwon-gu, Ansan-si, Gyeonggi-do	37.282181, 126.540257	KR049046	Ⅲ/Ⅱ
**Order Siphonostomatoida**							
Asterocheridae	*Asterocheres lilljeborgi*	Ec	LEGO-SIP002	Hyeonnae-myeon, Goseong-gun, Gangwon-do	38.492714, 128.427994	KR049050	Ⅵ/Ⅱ
Caligidae	*Lepeophtheirus salmonis*	Ec	LEGO-SIP012	Sacheonjin-ri, Sacheon-myeon, Gangneung-si, Gangwon-do	37.834125, 128.876106	KR049052	Ⅰ/Ⅱ
			LEGO-SIP012-1	Ganggu-ri, Ganggu-myeon, Yeongdeok-gun, Gyeongsangbuk-do	36.359633, 129.388916	KR049053	Ⅵ/Ⅱ
			-	Norway (Tjensvoll et al. 2006)	-	AY602766	-
Classification	Species	LS*	Voucher No.	Location	GPS	GenBank Acc. No.	PS**
	*Lepeophtheirus goniistii*	Ec	LEGO-SIP009	Gosan-ri, Hangyeong-myeon, Jeju-si, Jeju-do	33.307396, 126.163262	KR049054	Ⅵ/Ⅱ
	*Lepeophtheirus parviventris*	Ec	LEGO-SIP010	Gajin-ri, Jugwang-myeon, Goseong-gun, Gangwon-do	38.368210, 128.512220	KR049055	Ⅲ/Ⅱ
			-	Cananda (Jones and Prosperi-Porta 2011)	-	HM800840	-
	*Caligus fugu*	Ec	LEGO-SIP014	Gyeokpo-ri, Byeonsan-myeon, Buan-gun, Jeollabuk-do	35.623545, 126.467718	KR049056	Ⅵ/Ⅱ
	*Caligus punctatus*	Ec	LEGO-SIP006	Tappo-ri, Nambu-myeon, Geoje-si, Gyeongsangnam-do	34.713457, 128.627793	KR049057	Ⅵ/Ⅱ
	*Caligus hoplognathi*	Ec	LEGO-SIP021	Sinheung-ri, Jocheon-eup, Jeju-si, Jeju-do	33.548669, 126.640348	KR049058	Ⅵ/Ⅴ
	*Caligus quadratus*	Ec	LEGO-SIP020	Ganggu-ri, Ganggu-myeon, Yeongdeok-gun, Gyeongsangbuk-do	36.359633, 129.388916	KR049059	Ⅵ/Ⅱ
			-	Norway (Oines and Schram, 2008)	-	EF065619	-
Pandaridae	Pandaridae sp.	Ec	LEGO-SIP015	Ganggu-ri, Ganggu-myeon, Yeongdeok-gun, Gyeongsangbuk-do	36.359633, 129.388916	KR049060	Ⅰ/Ⅱ
Hatschekiidae	*Hatschekia japonica*	Ec	LEGO-SIP019	Gosan-ri, Hangyeong-myeon, Jeju-si, Jeju-do	33.307396, 126.163262	KR049051	Ⅷ/Ⅹ
Lernaeopodidae	*Haemobaphes pannosus*	Ec	LEGO-SIP016-8	Gajin-ri, Jugwang-myeon, Goseong-gun, Gangwon-do	38.368210, 128.512220	KR049061	Ⅵ/Ⅱ
			LEGO-SIP016	Ganggu-ri, Ganggu-myeon, Yeongdeok-gun, Gyeongsangbuk-do	36.359633, 129.388916	KR049062	Ⅰ/Ⅱ

* LS: Life style (adult): Fl = Free living, B = Benthic, As = Associated, Ec = Ectoparasitic.

** PS: PCR primer set used for amplifying *COI* from each individual.

Refer to Table 2 for primer sequences and lengths corresponding to the primer numbers I–X.

### Laboratory protocols

Ethanol-preserved specimens were rehydrated in distilled water for 5 hours before the procedure of DNA extraction. Genomic DNA was extracted using the QIAamp DNA micro kit (QIAGEN Co. Germany) in accordance with the manufacturer-recommended protocol with an exception that incubation with proteinase K was conducted overnight. For large specimens, the DNA was extracted with the Qiagen DNeasy Blood and Tissue Kit (QIAGEN Co. Germany).

The partial fragment of *COI* was amplified using the universal *COI* primer pair, HCO2198 and LCO1490 (Table 2) [33]. For specimens or species that did not amplify with this primer set, different specific forward and/or reverse primers were used (Table 1). The Bio-Rad Dyad Peltier thermal cycler was used to perform amplification using the following parameter: 2 min at 95°C, 34 cycles of 20 sec at 95°C, 40 sec at 42–48°C (Table 1) and 40 sec at 72°C, and 5 min at 72°C. PCR amplification was carried out in a 20μL reaction volume composed of 10–45 ng DNA extract, 0.75 mM of each deoxynucleotide, 0.25 mM of each forward and reverse primer, 3 mM MgCl_2_, 1 × PCR buffer, and 0.25 units of *Taq* DNA polymerase (Solgent Co., South Korea). PCR products were tested by electrophoresis on a QIAxcel Advanced (QIAGEN Co., Germany). The PCR products with the expected sized band were purified using QIAquick PCR purification kits (QIAGEN Co. Hilden Germany) along the manufacturer’s protocols. The PCR products were sequenced by the same set of primers used for the PCR amplifications, with ABI PRISM BigDye Terminator system and an ABI3700 automatic sequencer (Genotech Co., South Korea).

**Table 2:** Ten primers used for PCR amplification of partial *COI* from 133 individuals of 94 copepod species in this study. Primer sequences are given in 5′ to 3′ direction. Amplification difficulty caused by sequence variation of primer binding sites was resolved with mixed bases; R is a mixture of A and G, Y is a mixture of C and T, W is a mixture of A and T, D is a mixture of G, T and C. References are given for each primer.

No.	primer name	sequence (5'-3')	T_a_ (°C)	Reference
Ⅰ	LCO1490	GGT CAA CAA ATC ATA AAG ATA TTG G	48	Folmer et al. [33]
Ⅱ	HCO2198	TAA ACT TCA GGG TGA CCA AAA AAT CA	48	Folmer et al. [33]
Ⅲ	LCO1384	GGT CAT GTA ATC ATA AAG A	42	Machida et al. [34]
Ⅳ	cop-COI-1498F	GGG TGA CCA AAA AAT CAR AA	45	Bucklin et al. [28]
Ⅴ	cop-COI-2198R	AAY CAT AAA GAY ATY GGD AC	45	Bucklin et al. [28]
Ⅵ	cop-COX1+20	GAC TAA TCA TAA AGA TAT TGG TAC	45	Chang and Min, [35]
Ⅶ	HCO2612	AGG CCT AGG TGT ATW GGG AAA	42	Machida et al. [34]
Ⅷ	Coxf	GGT CCT GTA ATC ATA AAG AYA TYG G	45	Cheng et al. [36]
Ⅸ	Coxr1	GCG ACT ACA TAA TAA GTR TCR TG	45	Cheng et al. [36]
Ⅹ	Coxr2	TCT ATC CCA ACT GTA AAT ATR TGR TG	45	Cheng et al. [36]

### Sequence analyses

Chromatogram evaluation, editing, and assemblage were performed using BioEdit 7.0.9 [37]. The edited sequences were blasted against the GenBank nucleotide database (http://www.ncbi.nlm.nih.gov/). Subsequently, all sequences were aligned using Clustal X ver. 2.0.5 [38–39]. To check for the presence of pseudogenes or nuclear translocated mitochondrial sequences [40] in the *COI* dataset, sequences were carefully inspected for whether there were any stop codons or very divergent sequences [41]. The nucleotide sequences were translated to amino acids using EMBOSS Transeq (http://www.ebi.ac.uk/Tools/st/emboss_transeq/) based on the invertebrate mitochondrial genetic code. ClustalX ver. 2.0.5 was used to align each of these translated amino acids sequences with a gap opening of 10 and gap extension penalty of 0.2. The nucleotide sequence was then aligned with the amino acid alignment information using a scripted pipeline (convert-nuaa).

Genetic distances within species, genera, families and orders were calculated in MEGA 6 using Kimura two-parameter (K2P) models [42] for the alignments. Unrooted neighbor-Joining (NJ) trees were established using MEGA under the K2P evolutionary model with 1,000 bootstrapping replicates. The cluster analysis was shown in a radial tree topology, with node confidence values supported only by greater than 50% values.

## Results

The partial *COI* sequences from 133 individuals of 94 copepod species were determined and aligned. Although the size of the *COI* fragments amplified in the present study varied from 650 to 1,024 bp, the nucleotides at both ends were trimmed to only use high-quality, well matched data. A final sequences alignment of 575 bp was used in the analyses. Among the sequences, no sign of indels was revealed. Neither frame-shift mutations nor premature stop codons were detected during translation of the sequences into amino acids, supporting evidence that all of the sequences used were functional. Among the 575 bp of *COI*, 425 (74%) were polymorphic, of which 395 (69%) were parsimoniously informative. The average GC contents of all the sequences analyzed were 37.7%.

**Fig 2:**
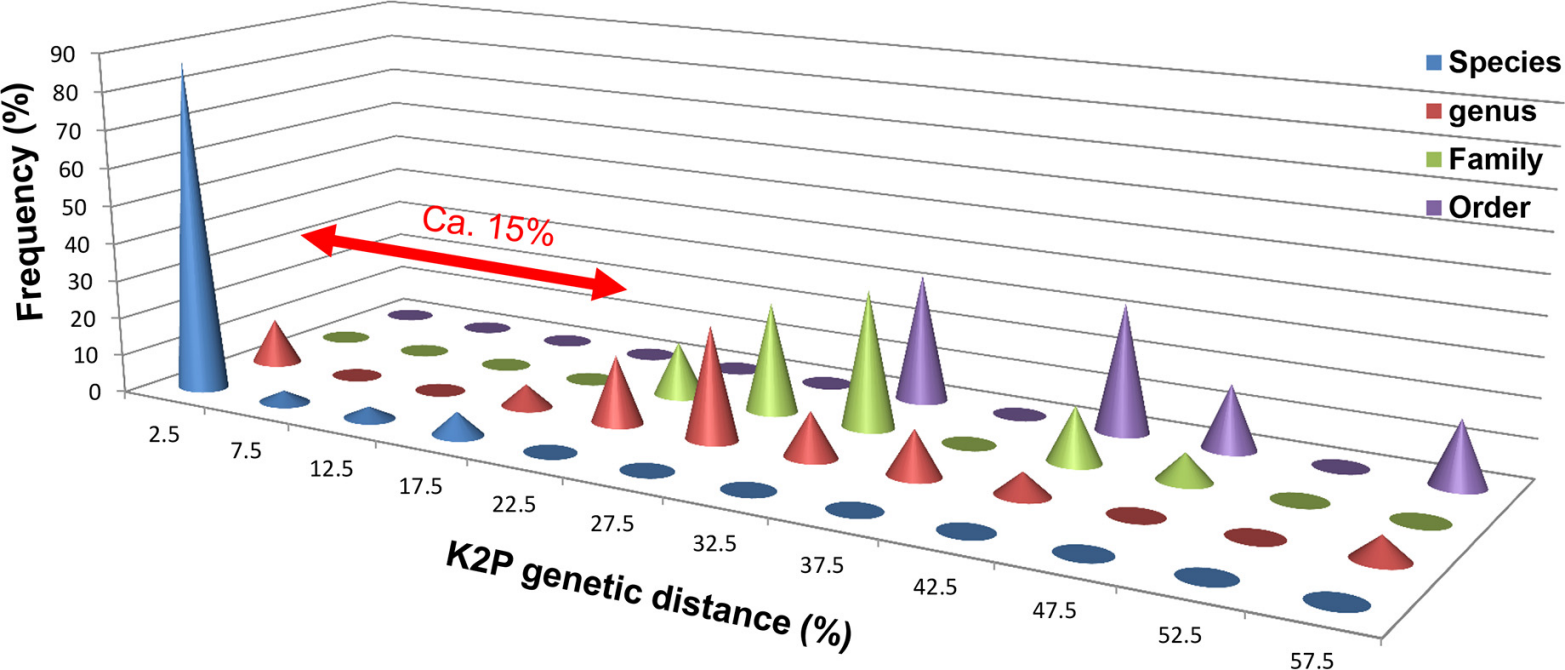
Distribution of pairwise genetic divergences estimated from nucleotide sequences of *COI* for 133 individuals of 94 copepod species including the six copepod orders based on the Kimura-2-parameter (K2P) distance matrix along four different taxonomic levels. The horizontal axis represents intervals of genetic distance in percentage and the vertical axis is the number of individuals associated with each distance interval. The flat box indicates zero value.

Mean divergences at various taxonomic levels are given in Table 3. As expected, the genetic divergence increases with higher taxonomic rank: 0.62% to 2.42% within species, 2.42% to 36.95% within genus, 13.00% to 56.94% within family, and 32.61% to 56.94% within order. Across copepod samples (*N* = 133), mean K2P divergence was 2.42% within species, 15.85% within genus, 24.22% within family, and 42.69% within order (Table 3). K2P distances within genus were highly variable, ranging from 2.42 (Siphonostomatoida) to 36.95 (Monstrilloida), though this type of comparison may not be reliable due to highly different sample sizes among copepod orders examined in this study (Table 3). Although these distance variability ranges were partially overlapped among specific, generic, familial and ordinal levels (Fig 2), it is likely that they were significantly different at a level sufficient to distinguish one copepod species from others.

**Table 3:** Mean genetic divergences at various taxonomic levels (species, genus, family, and order) inferred from nucleotide sequences of *COI* along the six copepod orders based on the Kimura-2-parameter (K2P) distances.

	Mean K2P distance (%)			
Order	Species	Genus	Family	Order
Calanoida	0.92	9.59	16.58	32.61
Cyclopoida	1.10	4.17	22.38	40.95
Monstrilloida	1.93	36.95	56.94	56.94
Harpacticoida	1.42	22.07	41.67	49.70
Poecilostomatoida	0.62	17.95	25.75	42.85
Siphonostomatoida	1.63	2.42	13.00	33.09
All groups	2.42	15.85	24.22	42.69

**Fig 3:**
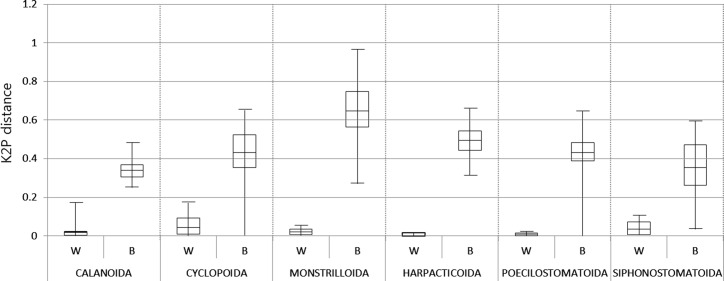
Boxplot distribution of pairwise genetic distances estimated from nucleotide sequences of *COI* for 133 individuals of 94 copepod species including the six orders based on the Kimura-2-parameter (K2P) distances. ‘W’ indicates genetic diversity within species and ‘B’ indicates that between species. The plot summarizes median (central bar), position of the upper and lower quartiles (central box), value of minimum (lower bar), and value of maximum (upper bar).

**Fig 4:**
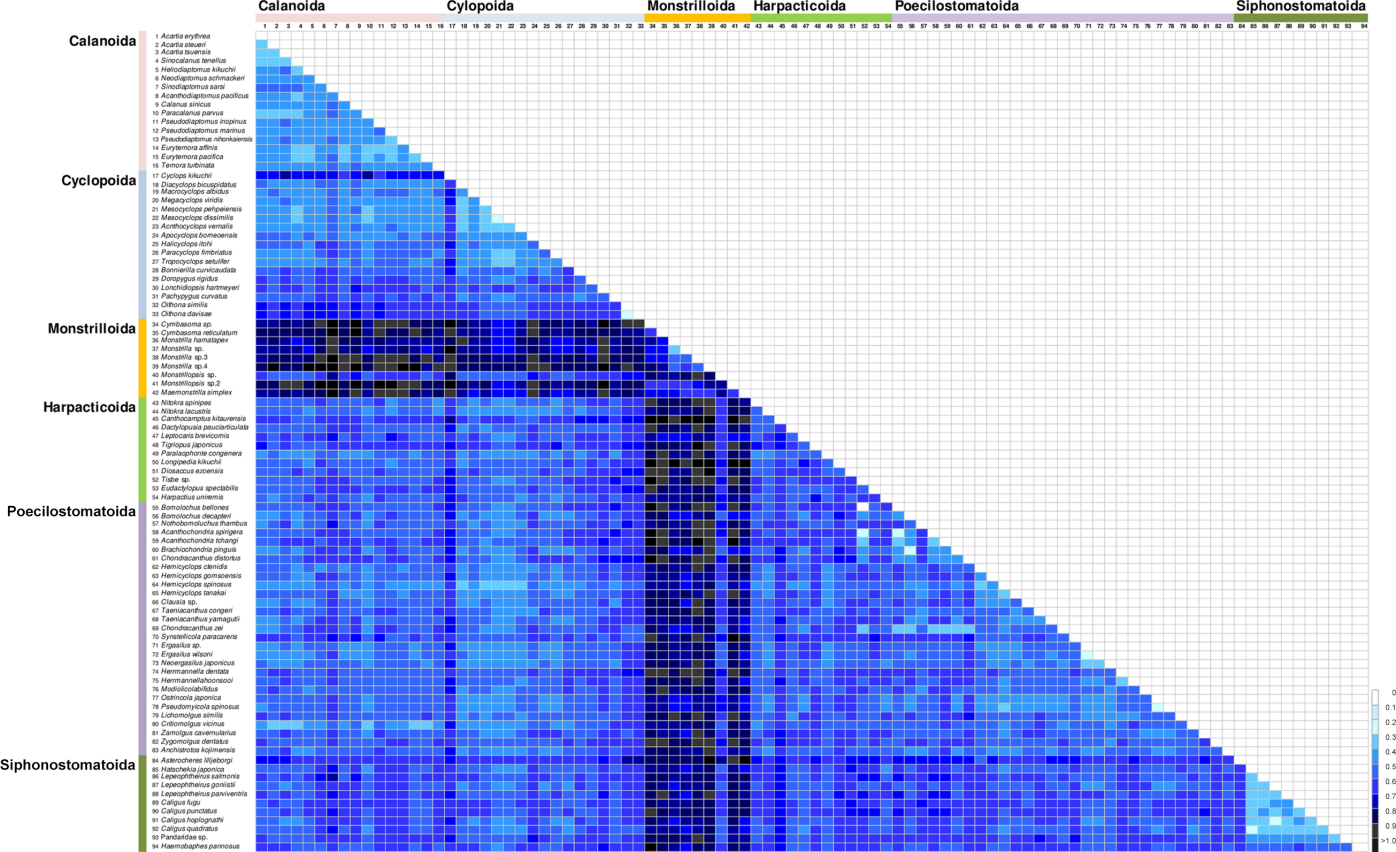
Color heatmap showing distribution of pairwise genetic distances estimated from nucleotide sequences of *COI* for 94 copepod species covering the six orders based on the Kimura-2-parameter (K2P) distances.

The *COI* genetic distances within and between species of the six copepod orders were summarized in Table 4 and Fig 3 (Refer to S1–S12 Tables and S1–S6 Figs). Within-species K2P distances ranged from 0.00% to 17.14% (Table 4), whereas between-species K2P distance from 0.17% to 96.53% (Table 4). There exists a clear gap with ca. 20 fold difference between the averages of within-species sequence divergence (2.42%) and between-species sequence divergence (42.79%) in *COI*, as shown in Table 4 and Fig 3, suggesting that the results of the present DNA barcoding of copepods could be effective in delimitating species. When we compared the *COI* barcoding data among 94 copepod species examined here, in most of them, a species could be distinguished from the others very clearly, only with the exceptions of four cases: *Mesocyclops dissimilis*–*Mesocyclops pehpeiensis* (0.26% K2P distance in percent) and *Oithona davisae*–*Oithona similis* (1.1%) in Cyclopoida, *Ostrincola japonica*–*Pseudomyicola spinosus* (1.5%) in Poecilostomatoida, and *Hatschekia japonica*–*Caligus quadratus* (5.2%) in Siphonostomatoida. A color heatmap representing the distribution of pairwise sequence divergence among 133 copepod individuals examined in this study showed comparatively and clearly greater values in Monstrilloida indicated by a darker color (Fig 4).

**Table 4:** Mean Kimura-2-parameter (K2P) distances within species and between species estimated from nucleotide sequences of *COI* for 133 individuals of 94 copepod species along the six different orders.

	Mean K2P distance (%)					
	Within-species			Between-species		
Order	Mean	Min	Max	Mean	Min	Max
Calanoida	2.17	0.00	17.14^1^	34.06	25.10	48.38
Cyclopoida	4.26	0.11	16.88^2^	43.28	0.26^3^	64.93
Monstrilloida	1.93	0.35	5.36	64.67	27.14	96.53
Harpacticoida	1.60	1.42	1.42	49.60	31.48	65.98
Poecilostomatoida	0.78	0.00	1.95	43.17	0.17^4^	64.44
Siphonostomatoida	3.37	0.52	10.32	35.45	3.60^5^	59.65
Averages	2.42	0.00	17.14	42.79	0.17	96.53

‘1’ and ‘2’ are the distance values (%) shown within *Paracalanus parvus* and *Macrocyclops albidus*.

‘3’ is a distance value (%) shown between *Mesocyclops pehpeinsis* and *Mesocyclops dissimilis*.

‘4’ is a distance value (%) shown between *Acanthochondria spirigera* and *Bomolochus bellones*.

‘5’ is a distance value (%) shown between *Lepeophtheirus goniistii* and *Caligus hoplognathi*.

**Fig 5:**
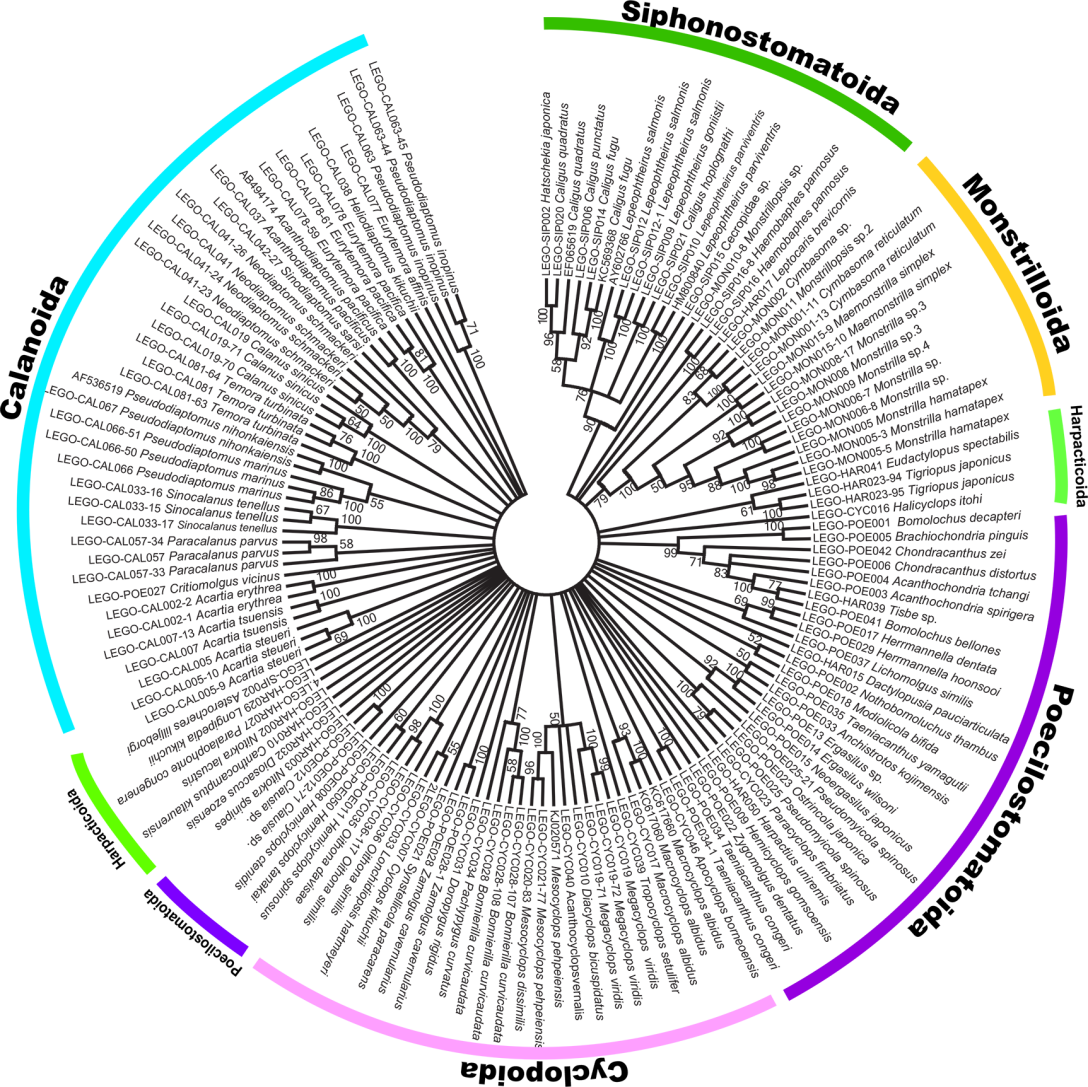
Unrooted neighbor-joining (NJ) tree reconstructed with nucleotide sequences of *COI* from 133 individuals of 94 species of copepods including the six different copepod orders. The analysis was done with Kimura-2-Parameter (K2P) distance matrix and 1,000 bootstrapping replicates. Branches supported with less than 50% bootstrap values were collapsed. The rate variation among sites was modeled with a gamma distribution. The asterisks indicate four species pairs, within each of which the two closely related species are not distinguished from each other based on the *COI* DNA barcoding marker.

The phylogenetic analysis of *COI* barcode sequences by a neighbor-joining method yielded an unrooted tree displayed in radial shape (Fig 5), which confidently showed a monophyletic clustering of individuals within a species in most of the copepod species examined here, albeit with the four exceptions indicated with asterisks (*) on the tree. In the four exceptional cases of *M*. *dissimilis*–*M*. *pehpeiensis* and *O*. *davisae*–*O*. *similis* in Cyclopoida, *O*. *japonica*–*P*. *spinosus* in Poecilostomatoida, and *H*. *japonica*–*C*. *quadratus* in Siphonostomatoida, the two closely related species were not clearly distinguished, respectively. Such exceptions are coincident with their lower between-species K2P distances inferred from the *COI* barcoding data. Also, each of the copepod orders with multiple genera and families formed a monophyletic clade. However, we could not find significant bootstrap support values in most basal nodes of the tree, suggesting a lack of phylogenetic signals of partial *COI* at higher taxonomic levels (Fig 5).

## Discussion

This study examined sequence variation of partial *COI* sequences and its utility as a DNA barcoding marker to identify and discriminate copepod species from six different copepod orders including Calanoida, Cyclopoida, Harpacticoida, Monstrilloida, Poecilostomatoida and Siphonostomatoida collected from the Korean Peninsula. Our results provide novel data with a wide sample range over the six copepod orders to confirm the validity of *COI* barcoding for copepod species identification. The ratio 21.9 of between-species to within-species sequence variation is more than twice of the threshold (= 10.0) proposed by Hebert et al. (2004) as a potential species’ boundary [43].

However, in the four unexpected cases of *M*. *dissimilis*–*M*. *pehpeiensis* and *O*. *davisae*–*O*. *similis* in Cyclopoida, *O*. *japonica*–*P*. *spinosus* in Poecilostomatoida, and *H*. *japonica*–*C*. *quadratus* in Siphonostomatoida, the *COI* marker did not provide clear-cut resolution of species identification. As an extreme example, three sequences determined from the two individuals of *Oithona similis* and one individual of *Oithona davisae* turned out to be almost identical (1.1% K2P distance in percent), while the two species are easily classified by distinctive morphological characters. Likewise, the other three cases had extremely lower between-species K2P distances (0.3–5.2%). Through further studies, it is necessary to be examined whether *COI* marker is appropriate for distinguishing such closely related species or not. On the other hand, *COI* sequences of *Paracalanus parvus* showed a relatively large difference among the three individuals within the species (S1 Table), although they formed a monophyletic groups (Fig 5). *P*. *parvus* has been known as a cosmopolitan copepod species and often confused with other morphologically similar species. Accordingly, multiple cryptic species could be involved with respect to the species, as mentioned in [44], and thus it is possible that this species may be a member of a species complex. If more detailed DNA barcoding work is done with multiple individuals from a variety of collection sites, the implication of high sequence similarity of *COI* shown in those copepod species could be clearly interpreted.

The present analyses revealed that the higher taxonomic rank of copepods, the more divergent the *COI* sequence variation is. Such tendency implied that the *COI* maker could be a powerful tool for confirmation of species identification as well as examination of copepod classification system based on morphological taxonomy of copepods (Tables 3 and 4, Figs 2–4).

Interestingly, between-species diversity (mean 64.67) of the order Monstrilloida and within-species diversity (mean 4.26) of the order Cyclopoida showed the highest values of genetic distances compared to those of the other orders (Table 4, Figs 3 and 4). Within-species diversity shown in cyclopoids may be due to much larger sample size and diversity examined here. High degree of between-species diversity shown in monstrilloid copepods may be closely related to their parasitic lifestyle. The order Monstrilloida is a unique and puzzling group, known as endo-parasites of polychaetes and mollusks during larval stages, though they become free-living and non-feeding plankton in their adult stage [45–46]. Parasitic montrilloid species often causes considerable difficulty in taxonomic classification due to their ambiguity of morphological characters: their mouthparts are highly reduced or nearly absent in their adult stage. One of the most important difficulties is to match monstrilloid males to their females. The only reliable method to link the sexes of a species is the confirmation of particular apomorphies shared by both sexes, by finding both sexes in the same host or as a pre-copulatory male-female pair in the plankton, or by using molecular identification [46]. Thus, the resultant divergence of monstrilloid *COI* sequences presented here could be helpful for understanding accelerated evolutionary rate of these parasitic copepod species, and also for designing suitable PCR primers to successfully amplify the *COI* barcode for molecular identification of monstrilloid copepod species.

It should be noted that one of the most fundamental problems encountered with DNA barcoding of copepods is the lack of a stable universal *COI* primer set and insufficient reference sequences. During the study, frequent PCR failures have repetitively occurred with some universal primers for most of copepods examined here. It may not be surprising if we take into account the fact that taxonomically broad copepods may have an enormous degree of *COI* sequence divergence.

Although DNA nucleotide sequences or deduced protein amino acid sequences from complete mitochondrial genomes have been frequently used to elucidate enigmatic arthropod phylogeny in higher taxonomical levels above order [47–53], it is generally known that the *COI* barcode marker, which is ca. 500–600 bp in length, does not contain enough phylogenetic signal for higher taxonomical levels. Rather, it can be more informative for questions related to population differentiation or cryptic speciation [18–19, 54–60]. Despite the weak resolution of the *COI* marker in familial- and ordinal-level phylogenetic relationships [60], the *COI*-based NJ tree (Fig 5) can be quite meaningful in terms of evidently showing the monophylies of most of the copepod species examined here as well as conveniently providing us with an overview of the *COI* barcoding results of 133 individuals from 94 copepod species including the six different orders at a glance.

It is known that mitochondrial genes evolve unusually rapidly in some copepods compared to those of other arthropods [61], with some closely related copepod species exhibiting unexpected gene order rearrangements [34, 62–64]. The previously known *COI* sequences are limited to a very small portion of copepods, which actually impedes the design of universal primers. Hopefully, as *COI* data of copepods grow, development of universal primers specific to copepods might be possible. Such group specific oligonucleotide sequences might be desirable to minimize contamination due to non-copepod PCR amplification, known as “the peril of universal primers” [65].

In summary, the present study including 133 individuals of 94 copepod species is the first attempt to establish a DNA barcoding system for a half dozen orders, which is the broadest survey yet reported in the literatures. It was found that a high degree of *COI* sequence divergence among most species was clearly sufficient for species identification of copepods in most cases. Thus, it is concluded that *COI* can serve as a standard, powerful molecular marker for DNA barcoding of copepod species, even though universal PCR primers specific to *COI* for copepods should be developed through further studies.

## Supporting Information

S1 Fig Distribution of pairwise genetic distances (= Kimura-2-parameter, K2P) estimated from *COI* nucleotide sequences of 16 calanoid species (N = 39). (PDF)

S2 Fig Distribution of pairwise genetic distances (= Kimura-2-parameter, K2P) estimated from *COI* nucleotide sequences of 17 cyclopoid species (N = 25). (PDF)

S3 Fig Distribution of pairwise genetic distances (= Kimura-2-parameter, K2P) estimated from *COI* nucleotide sequences of 9 monstrilloid species (N = 15). (PDF)

S4 Fig Distribution of pairwise genetic distances (= Kimura-2-parameter, K2P) estimated from *COI* nucleotide sequences of 12 harpacticoid species (N = 14). (PDF)

S5 Fig Distribution of pairwise genetic distances (= Kimura-2-parameter, K2P) estimated from *COI* nucleotide sequences of 29 poecilostomatoid species (N = 33). (PDF)

S6 Fig Distribution of pairwise genetic distances (= Kimura-2-parameter, K2P) estimated from *COI* nucleotide sequences of 11 siphonostomatoid species (N = 16). (PDF)

S1 Table Mean genetic distances within each species estimated from *COI* nucleotide sequences of 16 calanoid species (N = 39) based on Kimura-2-parameter distances. (PDF)

S2 Table Kimura-2-parameter pairwise distances between species estimated from *COI* nucleotide sequences of 16 calanoid species. (PDF)

S3 Table Mean genetic distances within each species estimated from *COI* nucleotide sequences of 17 cyclopoid species (N = 25) based on Kimura-2-parameter distances. (PDF)

S4 Table Kimura-2-parameter pairwise distances between species estimated from *COI* nucleotide sequences of 17 cyclopoid species. (PDF)

S5 Table Mean genetic distances within each species estimated from *COI* nucleotide sequences of 9 monstrilloid species (N = 15) based on Kimura-2-parameter distances. (PDF)

S6 Table Kimura-2-parameter pairwise distances between species estimated from *COI* nucleotide sequences of 9 monstrilloid species. (PDF)

S7 Table Mean genetic distances within each species estimated from *COI* nucleotide sequences of 12 harpacticoid species (N = 14) based on Kimura-2-parameter distances. (PDF)

S8 Table Kimura-2-parameter pairwise distances between species e estimated from *COI* nucleotide sequences of 12 harpacticoid species. (PDF)

S9 Table Mean genetic distances within each species estimated from *COI* nucleotide sequences of 29 poecilostomatoid species (N = 33) based on Kimura-2-parameter distances. (PDF)

S10 Table Kimura-2-parameter pairwise distances between species estimated from *COI* nucleotide sequences of 29 poecilostomatoid species. (PDF)

S11 Table Mean genetic distances within each species estimated from *COI* nucleotide sequences of 11 siphonostomatoid species (N = 16) based on Kimura-2-parameter distances. (PDF)

S12 Table Kimura-2-parameter pairwise distances between species estimated from *COI* nucleotide sequences of 11 siphonostomatoid species. (PDF)
