# Supplementary material for: DNA Barcoding of Metazoan Zooplankton Copepods from South Korea
Source: PLoS One. 2016 Jul 6;11(7):e0157307. doi: 10.1371/journal.pone.0157307 (PMC4934703; doi:10.1371/journal.pone.0157307)
Supplement: S1 Fig — (PDF) [file pone.0157307.s001.pdf]

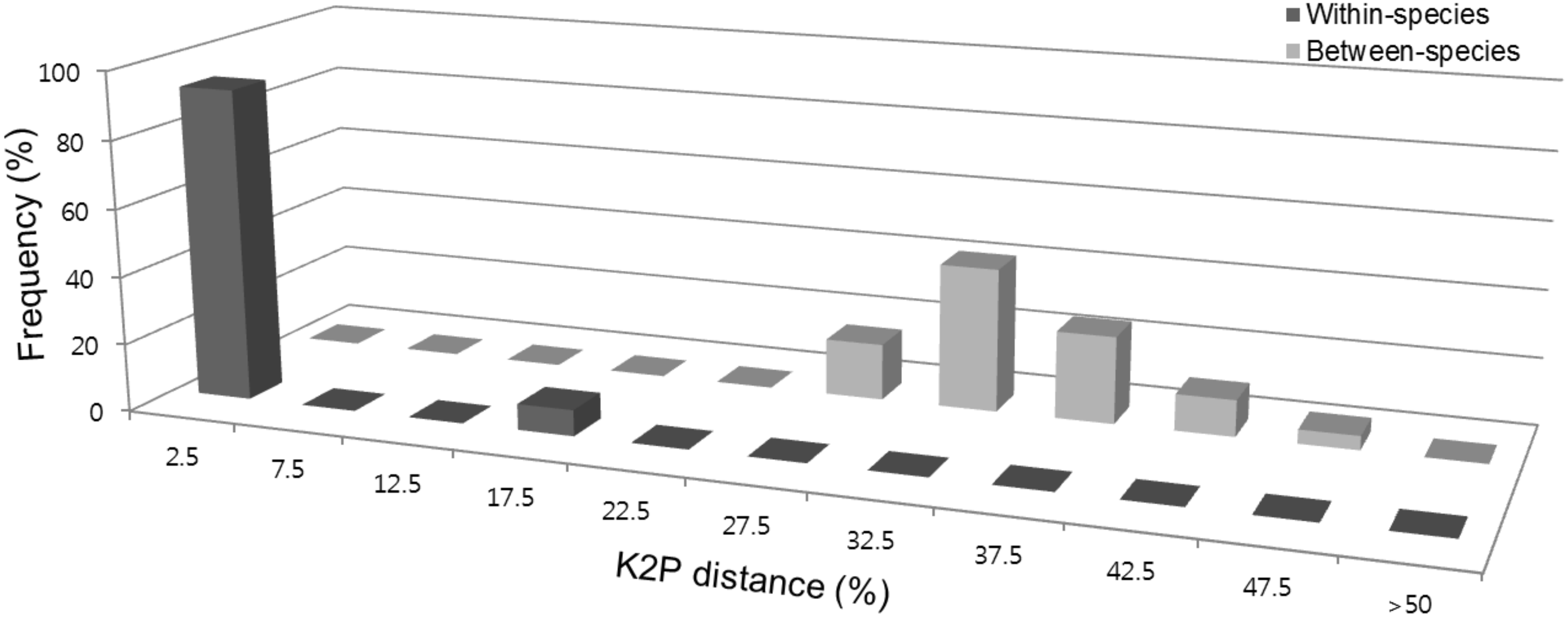

S1 Fig. Distribution of pairwise sequence divergence based on the Kimura-2-parameter for cytochrome *c* oxidase subunit I (*COI*) sequences of Calanoida.
