# Supplementary material for: DNA Barcoding of Metazoan Zooplankton Copepods from South Korea
Source: PLoS One. 2016 Jul 6;11(7):e0157307. doi: 10.1371/journal.pone.0157307 (PMC4934703; doi:10.1371/journal.pone.0157307)
Supplement: S6 Fig — (PDF) [file pone.0157307.s006.pdf]

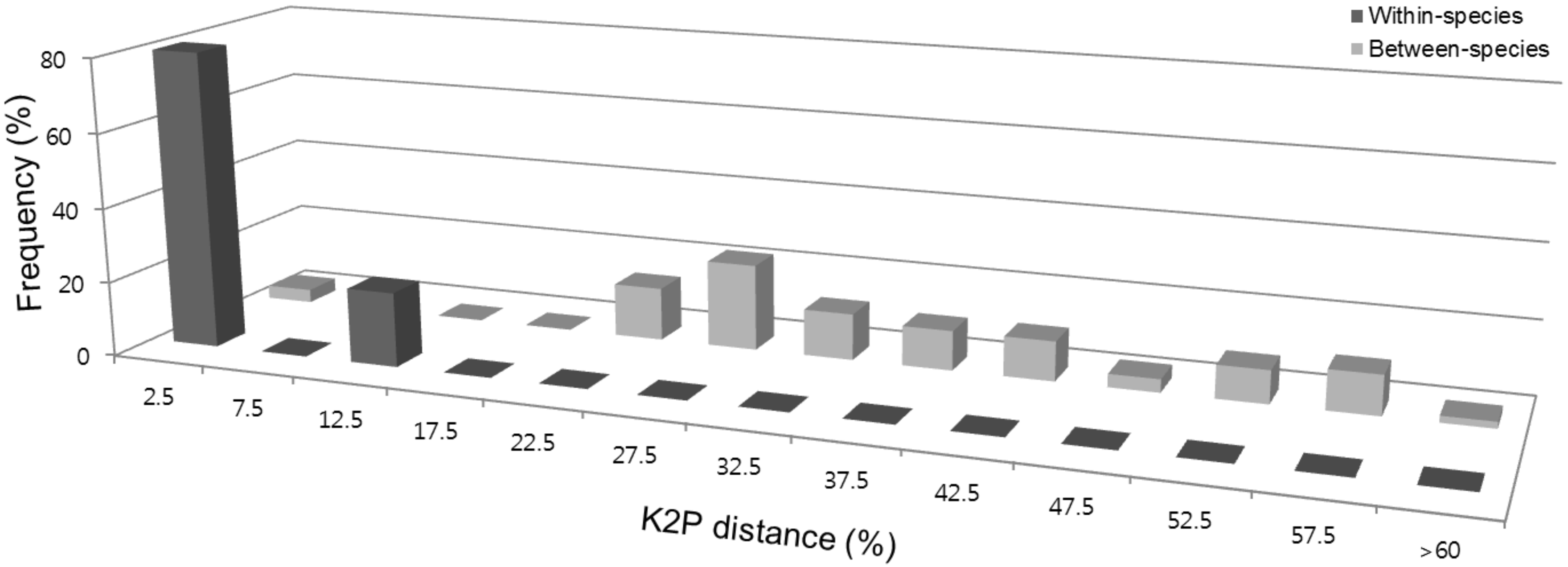

S6 Fig. Distribution of pairwise sequence divergence based on the Kimura-2-parameter for cytochrome *c* oxidase subunit I (*COI*) sequences of Siphonstomatoida.
