# Supplementary material for: DNA Barcoding of Metazoan Zooplankton Copepods from South Korea
Source: PLoS One. 2016 Jul 6;11(7):e0157307. doi: 10.1371/journal.pone.0157307 (PMC4934703; doi:10.1371/journal.pone.0157307)
Supplement: S1 Table — (PDF) [file pone.0157307.s007.pdf]

S1 Table. Mean genetic divergences (= Kimura-2-parameter distances) for the cytochrome oxidase *C* subunit 1 (*COI*) nucleotide sequences within species in the order Calanoida.

Species	Average	S. E.
Acartia erythrea	0.00	0.000
Acartia steueri	0.00	0.002
Acartia tsuensis	0.00	0.002
Sinocalanus tenellus	0.01	0.003
Heliodiaptomus kikuchii	-	-
Neodiaptomus schmackeri	0.01	0.004
Sinodiaptomus sarsi	-	-
Acanthodiaptomus pacificus	0.03	0.007
Calanus sinicus	0.00	0.002
Paracalanus parvus	0.17	0.017
Pseudodiaptomus inopinus	0.01	0.004
Pseudodiaptomus marinus	0.01	0.004
Pseudodiaptomus nihonkaiensis	0.01	0.003
Eurytemora affinis	-	-
Eurytemora pacifica	0.03	0.006
Temora turbinate	0.00	0.001
