# Supplementary material for: DNA Barcoding of Metazoan Zooplankton Copepods from South Korea
Source: PLoS One. 2016 Jul 6;11(7):e0157307. doi: 10.1371/journal.pone.0157307 (PMC4934703; doi:10.1371/journal.pone.0157307)
Supplement: S2 Table — (PDF) [file pone.0157307.s008.pdf]

S2 Table. Mean genetic divergences for the cytochrome oxidase *c* subunit 1 (*COI*) nucleotide sequences (Kimura-2-parameter [K2P] distances) of between-species among Calanoida.

		1	2	3	4	5	6	7	8	9	10	11	12	13	14	15	16
1	*Acartia erythrea*																
2	*Acartia steueri*	0.257															
3	*Acartia tsuensis*	0.292	0.266														
4	*Sinocalanus tenellus*	0.280	0.276	0.284													
5	*Heliodiaptomus kikuchii*	0.304	0.311	0.408	0.275												
6	*Neodiaptomus schmackeri*	0.329	0.357	0.395	0.350	0.338											
7	*Sinodiaptomus sarsi*	0.436	0.456	0.462	0.484	0.327	0.320										
8	*Acanthodiaptomus pacificus*	0.398	0.340	0.398	0.299	0.306	0.328	0.361									
9	*Calanus sinicus*	0.338	0.394	0.349	0.338	0.328	0.372	0.419	0.348								
10	*Paracalanus parvus*	0.292	0.251	0.292	0.257	0.318	0.351	0.466	0.312	0.323							
11	*Pseudodiaptomus inopinus*	0.360	0.370	0.435	0.361	0.309	0.362	0.394	0.347	0.378	0.383						
12	*Pseudodiaptomus marinus*	0.335	0.327	0.384	0.306	0.322	0.331	0.386	0.331	0.399	0.321	0.447					
13	*Pseudodiaptomus nihonkaiensis*	0.307	0.364	0.402	0.302	0.318	0.362	0.417	0.309	0.366	0.316	0.392	0.284				
14	*Eurytemora affinis*	0.303	0.314	0.321	0.264	0.265	0.324	0.358	0.296	0.329	0.285	0.299	0.291	0.340			
15	*Eurytemora pacifica*	0.316	0.308	0.359	0.275	0.264	0.374	0.415	0.286	0.351	0.299	0.321	0.296	0.326	0.263		
16	*Temora turbinate*	0.335	0.333	0.382	0.352	0.333	0.367	0.418	0.369	0.438	0.326	0.348	0.335	0.328	0.320	0.337	
