# Supplementary material for: DNA Barcoding of Metazoan Zooplankton Copepods from South Korea
Source: PLoS One. 2016 Jul 6;11(7):e0157307. doi: 10.1371/journal.pone.0157307 (PMC4934703; doi:10.1371/journal.pone.0157307)
Supplement: S3 Table — (PDF) [file pone.0157307.s009.pdf]

S3 Table. Mean genetic divergences for the cytochrome oxidase *c* subunit 1 (*COI*) nucleotide sequences (Kimura-2-parameter [K2P] distances) of within-species among Cyclopoida.

Species	Average	S. E.
Cyclops kikuchii	-	-
Diacyclops bicuspidatus	-	-
Macrocyclops albidus	16.88	0.018
Megacyclops viridis	1.18	0.004
Mesocyclops pehpeiensis	0.52	0.003
Mesocyclops dissimilis	-	-
Acnthocyclops vernalis	-	-
Apocyclops borneoensis	-	-
Halicyclops itohi	-	-
Paracyclops fimbriatus	-	-
Tropocyclops setulifer	-	-
Bonnierilla curvicaudata	1.29	0.004
Doropygus rigidus	-	-
Lonchidiopsis hartmeyeri	-	-
Pachypygus curvatus	-	-
Oithona similis	1.41	0.005
Oithona davisae	-	-
