# Supplementary material for: DNA Barcoding of Metazoan Zooplankton Copepods from South Korea
Source: PLoS One. 2016 Jul 6;11(7):e0157307. doi: 10.1371/journal.pone.0157307 (PMC4934703; doi:10.1371/journal.pone.0157307)
Supplement: S4 Table — (PDF) [file pone.0157307.s010.pdf]

S4 Table. Mean genetic divergences for the cytochrome oxidase *c* subunit 1 (*COI*) nucleotide sequences (Kimura-2-parameter [K2P] distances) of between-species among Cyclopoida.

		1	2	3	4	5	6	7	8	9	10	11	12	13	14	15	16	17
1	*Cyclops kikuchii*																	
2	*Diacyclops bicuspidatus*	0.579																
3	*Macrocyclops albidus*	0.649	0.342															
4	*Megacyclops viridis*	0.618	0.250	0.338														
5	*Mesocyclops pehpeiensis*	0.570	0.226	0.309	0.227													
6	*Mesocyclops dissimilis*	0.570	0.227	0.308	0.230	0.003												
7	*Acnthocyclops vernalis*	0.514	0.248	0.355	0.272	0.229	0.229											
8	*Apocyclops borneoensis*	0.589	0.389	0.383	0.343	0.341	0.343	0.353										
9	*Halicyclops itohi*	0.606	0.355	0.429	0.348	0.319	0.321	0.399	0.475									
10	*Paracyclops fimbriatus*	0.595	0.354	0.337	0.332	0.273	0.270	0.381	0.388	0.428								
11	*Tropocyclops setulifer*	0.641	0.345	0.360	0.312	0.293	0.295	0.324	0.371	0.384	0.328							
12	*Bonnierilla curvicaudata*	0.611	0.398	0.443	0.432	0.405	0.412	0.391	0.416	0.375	0.457	0.505						
13	*Doropygus rigidus*	0.587	0.479	0.466	0.425	0.398	0.396	0.460	0.510	0.448	0.431	0.505	0.416					
14	*Lonchidiopsis hartmeyeri*	0.507	0.479	0.518	0.487	0.513	0.510	0.468	0.476	0.528	0.600	0.553	0.419	0.439				
15	*Pachypygus curvatus*	0.550	0.386	0.403	0.429	0.389	0.391	0.420	0.457	0.440	0.411	0.437	0.331	0.428	0.462			
16	*Oithona similis*	0.577	0.530	0.555	0.485	0.484	0.484	0.496	0.526	0.561	0.548	0.518	0.527	0.533	0.537	0.533		
17	*Oithona davisae*	0.575	0.536	0.550	0.488	0.485	0.485	0.499	0.518	0.558	0.534	0.516	0.530	0.534	0.528	0.528	0.011	
