# Supplementary material for: DNA Barcoding of Metazoan Zooplankton Copepods from South Korea
Source: PLoS One. 2016 Jul 6;11(7):e0157307. doi: 10.1371/journal.pone.0157307 (PMC4934703; doi:10.1371/journal.pone.0157307)
Supplement: S5 Table — (PDF) [file pone.0157307.s011.pdf]

S5 Table. Mean genetic divergences for the cytochrome oxidase *c* subunit 1 (*COI*) nucleotide sequences (Kimura-2-parameter [K2P] distances) of within-species among Monstrilloida.

Species	Average	S. E.
Cymbasoma sp.	-	-
Cymbasoma reticulatum	1.22	0.004
Monstrilla hamatapex	0.94	0.003
Monstrilla sp.	5.36	0.010
Monstrilla sp.3	0.35	0.002
Monstrilla sp.4	-	-
Monstrillopsis sp.	-	-
Monstrillopsis sp.2	-	-
Maemonstrilla simplex	1.77	0.006
