# Supplementary material for: DNA Barcoding of Metazoan Zooplankton Copepods from South Korea
Source: PLoS One. 2016 Jul 6;11(7):e0157307. doi: 10.1371/journal.pone.0157307 (PMC4934703; doi:10.1371/journal.pone.0157307)
Supplement: S6 Table — (PDF) [file pone.0157307.s012.pdf]

S6 Table. Mean genetic divergences for the cytochrome oxidase *c* subunit 1 (*COI*) nucleotide sequences (Kimura-2-parameter [K2P] distances) of between -species among Monstrilloida.

		1	2	3	4	5	6	7	8	9
1	*Cymbasoma* sp.									
2	*Cymbasoma reticulatum*	0.566								
3	*Monstrilla hamatapex*	0.751	0.692							
4	*Monstrilla* sp.	0.664	0.634	0.271						
5	*Monstrilla* sp.3	0.695	0.663	0.419	0.426					
6	*Monstrilla* sp.4	0.809	0.748	0.497	0.508	0.463				
7	*Monstrillopsis* sp.	0.878	0.757	0.788	0.823	0.965	0.889			
8	*Monstrillopsis* sp.2	0.532	0.586	0.573	0.573	0.656	0.717	0.744		
9	*Maemonstrilla simplex*	0.683	0.690	0.600	0.565	0.593	0.595	0.701	0.562	
