# Supplementary material for: DNA Barcoding of Metazoan Zooplankton Copepods from South Korea
Source: PLoS One. 2016 Jul 6;11(7):e0157307. doi: 10.1371/journal.pone.0157307 (PMC4934703; doi:10.1371/journal.pone.0157307)
Supplement: S7 Table — (PDF) [file pone.0157307.s013.pdf]

S7 Table. Mean genetic divergences for the cytochrome oxidase *c* subunit 1 (*COI*) nucleotide sequences (Kimura-2-parameter [K2P] distances) of within-species among Harpacticoida.

Species	Average	S. E.
Nitokra spinipes	-	-
Nitokra lacustris	-	-
Canthocamptus kitaurensis	-	-
Dactylopusia pauciarticulata	-	-
Leptocaris brevicornis	-	-
Tigriopus japonicas	0.01	0.005
Paralaophonte congenera	-	-
Longipedia kikuchii	-	-
Diosaccus ezoensis	-	-
Tisbe sp.	-	-
Eudactylopus spectabilis	-	-
Harpactius uniremis	-	-
