# Supplementary material for: DNA Barcoding of Metazoan Zooplankton Copepods from South Korea
Source: PLoS One. 2016 Jul 6;11(7):e0157307. doi: 10.1371/journal.pone.0157307 (PMC4934703; doi:10.1371/journal.pone.0157307)
Supplement: S8 Table — (PDF) [file pone.0157307.s014.pdf]

S8 Table. Mean genetic divergences for the cytochrome oxidase *c* subunit 1 (*COI*) nucleotide sequences (Kimura-2-parameter [K2P] distances) of between-species among Harpacticoida.

		1	2	3	4	5	6	7	8	9	10	11	12
1	*Nitokra spinipes*												
2	*Nitokra acustris*	0.427											
3	*Canthocamptus kitaurensis*	0.492	0.446										
4	*Dactylopusia pauciarticulata*	0.452	0.476	0.543									
5	*Leptocaris brevicornis*	0.493	0.505	0.634	0.413								
6	*Tigriopus japonicus*	0.606	0.534	0.660	0.541	0.478							
7	*Paralaophonte congenera*	0.380	0.315	0.439	0.425	0.460	0.476						
8	*Longipedia kikuchii*	0.423	0.408	0.521	0.464	0.614	0.597	0.445					
9	*Diosaccus ezoensis*	0.400	0.484	0.524	0.551	0.539	0.569	0.414	0.442				
10	*Tisbe* sp.	0.558	0.485	0.587	0.474	0.508	0.515	0.512	0.576	0.547			
11	*Eudactylopus spectabilis*	0.500	0.490	0.568	0.414	0.452	0.441	0.472	0.542	0.524	0.440		
12	*Harpactius uniremis*	0.473	0.456	0.554	0.484	0.420	0.602	0.439	0.557	0.463	0.576	0.517	
