# Supplementary material for: DNA Barcoding of Metazoan Zooplankton Copepods from South Korea
Source: PLoS One. 2016 Jul 6;11(7):e0157307. doi: 10.1371/journal.pone.0157307 (PMC4934703; doi:10.1371/journal.pone.0157307)
Supplement: S9 Table — (PDF) [file pone.0157307.s015.pdf]

S9 Table. Mean genetic divergences for the cytochrome oxidase *c* subunit 1 (*COI*) nucleotide sequences (Kimura-2-parameter [K2P] distances) of within-species among Poecilostomatoida.

Species	Average	S. E.
Bomolochus bellones	-	-
Bomolochus decapteri	-	-
Nothobomoluchus thambus	-	-
Acanthochondria spirigera	-	-
Acanthochondria tchangi	-	-
Brachiochondria pinguis	-	-
Chondracanthus distortus	-	-
Hemicyclops ctenidis	-	-
Hemicyclops gomsoensis	-	-
Hemicyclops spinosus	-	-
Hemicyclops tanakai	-	-
Clausia sp.	0.52	0.003
Taeniacanthus congeri	0.00	0.000
Taeniacanthus yamagutii	-	-
Chondracanthus zei	-	-
Synstellicola paracarens	-	-
Ergasilus sp.	-	-
Ergasilus wilsoni	-	-
Neoergasilus japonicas	-	-
Herrmannella dentate	-	-
Herrmannella hoonsooi	-	-
Modiolicola bifidus	-	-
Ostrincola japonica	-	-
Pseudomyicola spinosus	1.95	0.006
Lichomolgus similis	-	-
Critiomolgus vicinus	-	-
Zamolgus cavernularius	0.00	0.000
Zygomolgus dentatus	-	-
Anchistrotos kojimensis	-	-
