# Supplementary material for: DNA Barcoding of Metazoan Zooplankton Copepods from South Korea
Source: PLoS One. 2016 Jul 6;11(7):e0157307. doi: 10.1371/journal.pone.0157307 (PMC4934703; doi:10.1371/journal.pone.0157307)
Supplement: S10 Table — (PDF) [file pone.0157307.s016.pdf]

S10 Table. Mean genetic divergences for the cytochrome oxidase *c* subunit 1 (*COI*) nucleotide sequences (Kimura-2-parameter [K2P] distances) of between-species among Poecilostomatoida.

		1	2	3	4	5	6	7	8	9	10	11	12	13	14	15	16	17	18	19	20	21	22	23	24	25	26	27	28	29
1	*Bomolochus bellones*																													
2	*Bomolochus decapteri*	0.353																												
3	*Nothobomoluchus thambus*	0.517	0.480																											
4	*Acanthochondria spirigera*	0.002	0.349	0.522																										
5	*Acanthochondria tchangi*	0.276	0.331	0.551	0.279																									
6	*Brachiochondria pinguis*	0.329	0.018	0.503	0.325	0.335																								
7	*Chondracanthus distortus*	0.308	0.362	0.570	0.304	0.313	0.348																							
8	*Hemicyclops ctenidis*	0.511	0.420	0.462	0.505	0.458	0.435	0.467																						
9	*Hemicyclops gomsoensis*	0.507	0.411	0.450	0.501	0.519	0.402	0.520	0.357																					
10	*Hemicyclops spinosus*	0.451	0.318	0.377	0.451	0.441	0.322	0.489	0.269	0.356																				
11	*Hemicyclops tanakai*	0.497	0.459	0.382	0.497	0.525	0.455	0.516	0.377	0.382	0.249																			
12	*Clausia* sp.	0.562	0.434	0.467	0.562	0.533	0.447	0.547	0.341	0.388	0.302	0.432																		
13	*Taeniacanthus congeri*	0.499	0.456	0.398	0.499	0.447	0.481	0.479	0.454	0.440	0.435	0.366	0.447																	
14	*Taeniacanthus yamagutii*	0.527	0.395	0.350	0.521	0.540	0.395	0.552	0.428	0.429	0.341	0.399	0.388	0.354																
15	*Chondracanthus zei*	0.298	0.233	0.450	0.294	0.290	0.239	0.278	0.423	0.425	0.348	0.443	0.420	0.469	0.443															
16	*Synstellicola paracarens*	0.530	0.551	0.522	0.525	0.525	0.541	0.623	0.490	0.480	0.466	0.466	0.511	0.500	0.474	0.454														
17	*Ergasilus* sp.	0.462	0.402	0.413	0.462	0.464	0.406	0.524	0.392	0.388	0.351	0.374	0.417	0.439	0.413	0.404	0.447													
18	*Ergasilus wilsoni*	0.451	0.398	0.412	0.451	0.459	0.402	0.518	0.401	0.371	0.362	0.391	0.412	0.430	0.394	0.401	0.449	0.025												
19	*Neoergasilus japonicus*	0.447	0.438	0.482	0.447	0.464	0.434	0.525	0.415	0.349	0.376	0.412	0.375	0.484	0.443	0.390	0.418	0.249	0.246											
20	*Herrmannella dentata*	0.501	0.523	0.472	0.507	0.560	0.523	0.571	0.420	0.531	0.429	0.468	0.419	0.454	0.444	0.490	0.546	0.452	0.444	0.450										
21	*Herrmannella hoonsooi*	0.470	0.500	0.490	0.470	0.533	0.507	0.582	0.411	0.470	0.386	0.429	0.418	0.447	0.479	0.484	0.492	0.388	0.368	0.425	0.268									
22	*Modiolicola bifidus*	0.518	0.471	0.342	0.512	0.540	0.487	0.486	0.406	0.400	0.379	0.412	0.406	0.429	0.436	0.423	0.477	0.425	0.437	0.379	0.415	0.455								
23	*Ostrincola japonica*	0.461	0.352	0.442	0.466	0.501	0.367	0.561	0.389	0.367	0.302	0.388	0.386	0.388	0.394	0.359	0.464	0.323	0.312	0.317	0.434	0.428	0.404							
24	*Pseudomyicola spinosus*	0.463	0.339	0.448	0.458	0.502	0.357	0.544	0.389	0.363	0.293	0.390	0.384	0.391	0.388	0.354	0.465	0.328	0.320	0.327	0.433	0.421	0.407	0.015						
25	*Lichomolgus similis*	0.495	0.534	0.471	0.490	0.590	0.528	0.644	0.422	0.484	0.431	0.447	0.422	0.503	0.460	0.493	0.453	0.396	0.384	0.366	0.357	0.374	0.426	0.437	0.428					
26	*Critiomolgus vicinus*	0.450	0.406	0.464	0.445	0.555	0.414	0.496	0.434	0.455	0.361	0.443	0.418	0.513	0.468	0.359	0.482	0.410	0.418	0.378	0.464	0.503	0.470	0.411	0.401	0.474				
27	*Zamolgus cavernularius*	0.494	0.447	0.428	0.489	0.460	0.448	0.547	0.402	0.480	0.378	0.492	0.501	0.520	0.475	0.407	0.424	0.472	0.477	0.421	0.476	0.464	0.399	0.359	0.361	0.423	0.425			
28	*Zygomolgus dentatus*	0.476	0.546	0.563	0.470	0.562	0.540	0.526	0.444	0.415	0.487	0.407	0.484	0.465	0.497	0.512	0.464	0.457	0.447	0.380	0.507	0.482	0.464	0.454	0.463	0.444	0.491	0.573		
29	*Anchistrotos kojimensis*	0.464	0.383	0.353	0.469	0.511	0.379	0.487	0.413	0.396	0.321	0.363	0.437	0.401	0.303	0.419	0.493	0.397	0.397	0.419	0.437	0.459	0.405	0.365	0.368	0.480	0.440	0.483	0.418	
