# Supplementary material for: DNA Barcoding of Metazoan Zooplankton Copepods from South Korea
Source: PLoS One. 2016 Jul 6;11(7):e0157307. doi: 10.1371/journal.pone.0157307 (PMC4934703; doi:10.1371/journal.pone.0157307)
Supplement: S11 Table — (PDF) [file pone.0157307.s017.pdf]

S11 Table. Mean genetic divergences for the cytochrome oxidase *c* subunit 1 (*COI*) nucleotide sequences (Kimura-2-parameter [K2P] distances) of within-species among Siphonostomatoida.

Species	Average	S. E.
Asterocheres lilljeborgi	-	-
Hatschekia japonica	-	-
Lepeophtheirus salmonis	4.06	0.007
Lepeophtheirus goniistii	-	-
Lepeophtheirus parviventris	1.41	0.005
Caligus fugu	0.52	0.003
Caligus punctatus	-	-
Caligus hoplognathi	-	-
Caligus quadratus	10.32	0.015
Pandaridae sp.	-	-
Haemobaphes pannosus	0.52	0.003
