# Supplementary material for: DNA Barcoding of Metazoan Zooplankton Copepods from South Korea
Source: PLoS One. 2016 Jul 6;11(7):e0157307. doi: 10.1371/journal.pone.0157307 (PMC4934703; doi:10.1371/journal.pone.0157307)
Supplement: S12 Table — (PDF) [file pone.0157307.s018.pdf]

S12 Table. Mean genetic divergences for the cytochrome oxidase *c* subunit 1 (*COI*) nucleotide sequences (Kimura-2-parameter [K2P] distances) of within-species among Siphonostomatoida.

		1	2	3	4	5	6	7	8	9	10	11
1	*Asterocheres lilljeborgi*											
2	*Hatschekia japonica*	0.544										
3	*Lepeophtheirus salmonis*	0.555	0.263									
4	*Lepeophtheirus goniistii*	0.535	0.216	0.260								
5	*Lepeophtheirus parviventris*	0.596	0.249	0.293	0.263							
6	*Caligus fugu*	0.534	0.244	0.275	0.320	0.371						
7	*Caligus punctatus*	0.556	0.213	0.327	0.284	0.357	0.231					
8	*Caligus hoplognathi*	0.555	0.214	0.272	0.036	0.276	0.315	0.280				
9	*Caligus quadratus*	0.545	0.052	0.264	0.213	0.270	0.254	0.228	0.207			
10	Pandaridae sp.	0.591	0.316	0.360	0.313	0.348	0.379	0.366	0.331	0.299		
11	*Haemobaphes pannosus*	0.587	0.423	0.409	0.408	0.425	0.510	0.489	0.409	0.402	0.486	
